# The Effects of COVID-19 on the Eating Habits of Children and Adolescents in Italy: A Pilot Survey Study

**DOI:** 10.3390/nu13082641

**Published:** 2021-07-30

**Authors:** Roberta Pujia, Yvelise Ferro, Samantha Maurotti, Janin Khoory, Carmine Gazzaruso, Arturo Pujia, Tiziana Montalcini, Elisa Mazza

**Affiliations:** 1 Department of Medical and Surgical Science, University Magna Grecia, 88100 Catanzaro, Italy; roberta.puj@gmail.com (R.P.); samabiotec@yahoo.it (S.M.); pujia@unicz.it (A.P.); elisamazza@unicz.it (E.M.); 2 Department of Health Science, University Magna Grecia, 88100 Catanzaro, Italy; 3 Nutrition Unit, University Magna Grecia, 88100 Catanzaro, Italy; janin.khoory@libero.it; 4 Department Biomedical Science for Heath, University of Milan, 20133 Milano, Italy; c.gazzaruso@gmail.com; 5 Research Center for the Prevention and Treatment of Metabolic Diseases, University Magna Grecia, 88100 Catanzaro, Italy; tmontalcini@unicz.it; 6 Department of Clinical and Experimental Medicine, University Magna Græcia, 88100 Catanzaro, Italy

**Keywords:** COVID-19, eating habits, body weight gain, children, adolescents, obesity

## Abstract

Nutrition during childhood and adolescence is very important for growth and can have long-term health implications. The COVID-19 lockdown caused significant changes in everyday life, including in children and adolescents. This study evaluated the effects of the first COVID-19 lockdown on eating habits and body weight in children and adolescents. An online cross-sectional survey was carried out among the parents of children (5–9 years) and adolescents (10–14 years) living in Italy. The online self-administered questionnaire included demographic and anthropometric data (reported weight and height) weight and dietary habit changes during the COVID-19 lockdown in Italy (March to June 2020). A total of 439 participants were included. We found a change in eating habits with an increase in consumption of sweet packaged snacks (34%) and processed meat (25%), as well as bread, pizza and bakery products (47%). We also found an increase in vegetable, fresh fruit and legume intake (19%), and a reduction in sweet beverage and candy intake. A total of 59.7% of the participants reported body weight gain, with adolescents gaining more than children (67% vs. 55%, *p* = 0.010, respectively). In children, body weight gain was associated with a change in body height and increased consumption of dairy products and sweet packaged snacks, while in adolescents it was associated with an increased intake of comfort foods and processed meat. Our data highlighted the need to carefully monitor eating behaviors to avoid the establishment of unhealthy eating habits and prevent obesity in children and adolescents during periods of self-isolation.

## 1. Introduction

The coronavirus (COVID-19) outbreak is considered the greatest threat to global health in recent years. This condition led to a public health emergency of international concern, so much so that on 11 March 2020, the World Health Organization (WHO) declared COVID-19 as a pandemic [1]. In order to contain the COVID-19 pandemic, the Italian government [2], in agreement with other countries of the world, as indicated by UNESCO [3], put in place rigorous containment measures. This “Stay-at-Home” decree [2] changed the lifestyles and habits of the Italian population during the lockdown [4,5].

School closures, physical distance and isolation have had a strong impact on the lives of children and young people. In some countries, children were allowed to play sports or walks outdoor, while in Italy such activities were prohibited. From both emotional and physical perspectives, health professionals warned of the threat that this lockdown could be for the well-being of children [6,7].

Research carried out in China suggested that the COVID-19 lockdown generated feelings of worry, fear, loneliness, sadness and stress in individuals aged 3 to 18 [8,9,10,11]. An Italian study, conducted during the first COVID-19 lockdown, which involved children between the ages of 4 and 10, also found that children were showing new fears, increased irritability, intolerance to rules, nervousness, tantrums and excessive demands, as well as mood changes and sleep problems [12]. Therefore, children and adolescents were very susceptible to the impact of the lockdown, and their psycho-physical well-being was negatively affected. Other reasons to be concerned about housebound children and adolescents are the consequent changes in eating habits. Previous studies have reported that young individuals have difficulty with weight-control lifestyle programs while at home compared to when they are in their usual school curriculum [13]. Furthermore, recent observations show that during the lockdown, many households consumed more ultra-processed and calorie-dense foods than usual [14].

To the best of our knowledge, only one study in Italy has evaluated the change in eating habits in children during the lockdown [15]. The authors studied a small sample of obese adolescents and children in northern Italy between the ages of 6 and 18 [15].

The aim of our study was thus to evaluate the changes in eating habits in adolescents and children in Italy, and their impact on body weight, during the first lockdown.

## 2. Materials and Methods

This cross-sectional study was carried out in Italy to explore the effects of first COVID-19 lockdown (March to June 2020) on the changes in eating habits in Italian adolescents and children. For this study, we performed an online survey questionnaire using the Google Forms tool. Eating habits are different between children and adolescents, therefore we decided to classify the participants into two groups, children (5–9 years) and adolescents (10–14 years). To have a more representative sample of Italian children and adolescents, the questionnaire was distributed in two different cities in the country, one in the north and one in the south. The questionnaire was distributed electronically to the parents of adolescents and children living in Brescia (Lombardy, Italy) (in Italian, retrievable at https://docs.google.com/forms/d/11H8C1o8qvQ4oATcavAkjQbgpx3_1svI9CPITrygx0Uo/edit) (accessed on 20 October 2020) through pediatricians, and to parents of children and adolescents living in Catanzaro (Calabria, Italy) (in Italian, retrievable at https://forms.gle/FXNCg8FXxEVwXy9D8) (accessed on 11 January 2021) via a private social network (Facebook) by dieticians and nutritionists. The items were the same for participants living in Lombardy as well as for those in Calabria. The pediatricians who collaborated with this research enrolled children and adolescents during outpatient medical examinations by asking for their parents’/caregivers’ consent to participate in the study and to provide an email address. About 600 emails were sent to parents/caregivers, 250 of which participated in the online dietary survey. The rest of the participants responded to a link sent via Facebook.

The aim of the questionnaires was to assess whether, in the first lockdown, there was an increase or reduction in the intake of selected foods, as well as soft drink consumption and weight, observed during COVID-19 quarantine. In particular, the questionnaire was designed to collect demographic information (age, gender, changes in physical activity); anthropometric data (reported height and body weight); and information about eating habits (the frequency of milk, dairy, vegetable, legume, fruits, meat, fish, egg, pasta, rice, bread, pizza, sweet, oil, margarine, butter and soft beverage consumption before and during the lockdown). We used the same method, but a modified version, of the questionnaire of the Italian council for research in agriculture and agrarian economics (CREA), which was previously used in a survey [16] (retrievable at https://docs.google.com/forms/d/18lydVFUhLJYqxtsfy9CYsgRGnmYDG0XVpo3PklEi_VM/prefill) (accessed on 22 September 2020).

To validate our modified version, we used the technique suggested by Chopra et al. [17]. In particular, we performed a factor analysis via Horns parallel analysis for principal components on the questionnaires, using varimax rotation [17]. An eigenvalue of 1 was used as a cut-off for determining the number of factors. Overall, the total percentage of variance was 53.5%. Cronbach’s α was used to evaluate internal consistency (i.e., the extent to which elements of the instrument measure the same thing). A good internal consistency is represented by a value >0.70 and our questionnaire reached a Cronbach’s α value of 0.86.

This type of online research is a recommended approach to quickly reach a specific group of subjects, guaranteeing their safety under a pandemic [18,19]. The questionnaires were made available via online social media for the period between 10 September 2020 and 19 April 2021.

### 2.1. Study Participants

Parents with children aged 5–14 years were invited to participate. The following inclusion criteria were applied: being able to complete the study questionnaire in Italian, having children or adolescents aged 5–14 years of either gender and providing a consent form. These criteria were verified by the answers given to the corresponding survey questions. The survey was completed by 439 parents of children and adolescents. According to the difference between the values of each eating behavior before and during the quarantine, each parameter was categorized as “decreased” (i.e., if before-lockdown value of food consumption was higher than during-lockdown value), “stable” (i.e., same values of food intake before and during the lockdown) or “increased” (i.e., if before-lockdown value of food consumption was lower than during-lockdown value).

Changes in body weight and height for each child/adolescent were calculated as the difference between self-reported weight and height from the period before and during the lockdown. According to the change in body weight before and during the first COVID-19 lockdown, children/adolescents were categorized into two groups: 1. increased body weight (if their weight was higher than before the lockdown); 2. not increased body weight (if their weight was stable or reduced). The body mass index (BMI) was calculated as weight (kg)/height (m)^2^, and BMI change as the difference between BMI before and after the lockdown. We used the definition of obesity proposed by the Childhood Obesity Working Group of the International Obesity Task Force [20], which is linked to the widely used adult obesity cut off point of 30 kg/m^2^.

### 2.2. Ethical Considerations

Anonymity of the children and adolescents was guaranteed by the Google platform. Participation in the survey was voluntary and no recompense was provided. The research protocol was approved by the Local Ethics Committee in the Calabria Region—Central Area (127/2020/CE approved 16 April 2020). All parents signed an informed consent form prior to their participation in the study. The investigation conforms to the principles outlined in the Declaration of Helsinki.

### 2.3. Statistical Analysis

We enrolled a convenience sample. However, we calculated that to detect dietary intake variations of at least 15–20% in children as well as adolescents, with 80% power with a two-sided level of significance of 0.05, a minimum of 62 individuals for each group was required. After closing the survey and stopping data collection, the final database was downloaded as a Microsoft Excel sheet and data were analyzed immediately thereafter. Data are reported as mean ± standard deviation (SD) for continuous variables. Categorical variables are presented as absolute (*n*) and relative (%) frequencies. Participants were classified into two age groups: children with an age of 5 to 9 years, and adolescents with an age of 10 to 14 years. A chi square test was performed to analyze the proportion of the population who changed their eating habits according to age group and body weight changes. Mean values of anthropometric parameters before and during the quarantine were compared using a Student’s *t*-test for unpaired data. According to age group, a Pearson’s correlation was used to identify all the confounding variables correlated with the body weight change given that the continuous variables were distributed normally. A similar analysis was also performed with BMI change. A stepwise logistic regression analysis was utilized to assess which variables were associated with body weight change. The variables were selected among those with *p* < 0.1 in the Pearson’s correlation analysis. Significant differences were assumed to be present at *p* < 0.05 (two-tailed). All comparisons were performed using SPSS 25.0 for Windows (IBM Corporation, New York, NY, USA).

## 3. Results

We obtained a total of 439 dietary intake assessments from children and adolescents, with boys representing 56% of the population.The main characteristics of the participants are presented in Table 1. The average age of the sample was 8.8 ± 3 years, and the majority of the participants (58.1%) were 5–9 years old.

### 3.1. Reported Change in Body Weight

**Figure 1:**
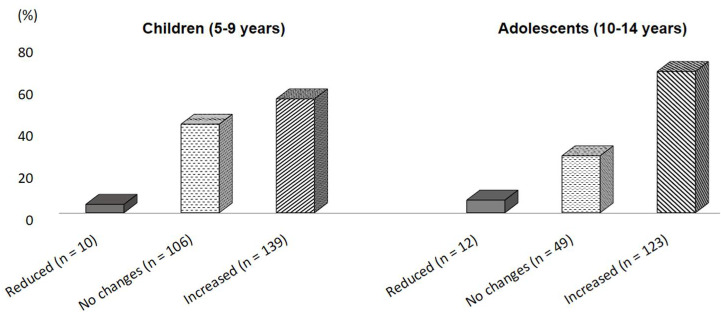
Proportion of the population who changed in body weight during the first lockdown, according to age group.

The basal body weight of the children/adolescents was 32.2 ± 13 kg. The first lockdown did not substantially change the body weight of 155 (35.3%) participants, while 262 (59.7%) of them reported a weight gain (Table 1). In particular, 16.2% children/adolescents increased their body weight by more 3 kg with no significant difference between genders (63% vs. 56%, *p* = 0.17). Furthermore, we found a greater weight gain in adolescents than children (67% vs. 55%, *p* = 0.010). Body weight changes in the population according to age group are shown in Figure 1. An analysis of subgroup, after excluding obese subjects, did not modify this finding (73% vs. 58%, *p* = 0.004, data not shown).

### 3.2. Eating Habits during Lockdown

**Figure 2:**
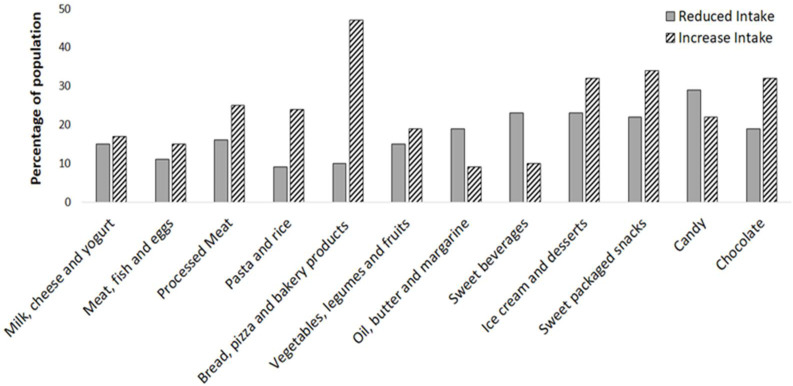
Proportion of the population who changed their eating habits during the first COVID-19 lockdown.

Dietary habits during the first COVID-19 lockdown are reported in Figure 2. A significant number of parents reported that their children/adolescents changed their eating habits during the lockdown (Figure 2).

#### 3.2.1. Comfort Food

An increase in “comfort food” consumption, notably chocolate (32%), sweet packaged snacks (34%) and ice cream and desserts (32%), but also pasta and rice (24%), as well as bread, pizza and bakery products (47%), was found. Conversely, 29% and 23% of children/adolescents decreased their consumption of candies and sweetened beverages, respectively.

#### 3.2.2. Vegetable, Legumes and Fruits

Interestingly, 19% of responders increased their consumption of vegetables, legumes and fresh fruit during the first lockdown.

#### 3.2.3. Meat Products, Milk, Cheese, Yogurt and Fats

The data showed a greater increase in the consumption of meat, fish and eggs (15%) but also of processed meat (25%). Consumption of milk and dairy products increased during the first COVID-19 lockdown (17%). There was also a decrease in the intake of butter, margarine and oils (19%).

### 3.3. Changes in Eating Habits According to Body Weight Change during Lockdown

Table 2 shows the demographic and anthropometric characteristics, and the changes in lifestyle and eating habits of participants during the first COVID-19 lockdown according to age group and changes in body weight.

The table highlights that compared to the children with stable or reduced body weight, in the group of subjects aged 5 to 9 years, the children who gained body weight were older and reported an increase in milk/cheese/yogurt; processed meat; pasta/rice; bread/pizza/baked goods; oil/butter/margarine; ice cream/dessert; packaged sweet snacks; candy and chocolate consumption (Table 2). Furthermore, subjects with a greater body weight gain also had a greater change in height than children with stable or low body weight (Table 2).

Compared to those with stable or reduced body weight, in the group of subjects aged 10 to 14 years, the adolescents who gained body weight had a more sedentary lifestyle and greater variation in height (Table 2), and the adolescents with body weight gain reported increased consumption of milk/cheese/yogurt; processed meat; bread/pizza/baked goods; and soft drinks (Table 2).

### 3.4. Predictors of Body Weight Change

In children, Pearson’s correlation showed that body weight gain correlated with age (r = 0.18; *p* = 0.003), change in body height (r = 0.32; *p* = 0.001) and milk/cheese/yogurt (r = 0.27; *p* < 0.001), processed meat (r = 0.28; *p* < 0.001), pasta/rice (r = 0.23; *p* < 0.001) and bread/pizza/bakery product consumption (r = 0.15; *p* = 0.01). Furthermore, body weight gain also correlated with oil/butter/margarine (r = 0.14; *p* = 0.02), ice cream/dessert (r = 0.21; *p* = 0.001), packaged sweet snack (r = 0.23; *p* < 0.001), candy (r = 0.13; *p* = 0.03) and chocolate consumption (r = 0.21; *p* = 0.001). Basal BMI did not correlate with weight gain (*p* = 0.27). A Pearson’s correlation showed that BMI gain also correlated with the same food groups described above in children.

However, in adolescents, Pearson’s correlation showed that the body weight gain correlated with age (r = 0.13; *p* = 0.05), body height at baseline (r = 0.26; *p* = 0.02), physical activity change (r = 0.25; *p* < 0.001) and milk/cheese/yogurt (r = 0.15; *p* = 0.03), meat/fish/egg (r = 0.12; *p* = 0.08) and processed meat consumption (r = 0.18; *p* = 0.01). Furthermore, body weight gain also correlated with bread/pizza/bakery product (r = 0.18; *p* = 0.01), ice cream/dessert (r = 0.13; *p* = 0.06), packaged sweet snack (r = 0.23; *p* = 0.04), candy (r = 0.12; *p* = 0.08), chocolate (r = 0.13; *p* = 0.06) and sweet beverage consumption (r = 0.16; *p* = 0.02). Basal BMI did not correlate with weight gain (*p* = 0.31). Pearson’s correlation also showed that BMI gain correlated only with processed meat (*p* = 0.08) and candy consumption (*p* = 0.03) in adolescents.

In the logistic regression analysis (Table 3), the body weight gain remained associated with height change, and consumption of milk/cheese/yogurt and sweet packaged snacks in the children’s group. Finally, in the adolescents’ group, logistic regression analysis showed an association between body weight gain and intake of processed meat and bread/pizza/bakery products (Table 3).

## 4. Discussion

Our study evaluated the effects of the first COVID-19 lockdown on the eating habits of adolescents and children. To the best of our knowledge, this is the first study using a representative sample of children and adolescents from northern and southern Italy. Although spending all their time at home could potentially improve eating behaviors since children and adolescents could be more restricted in their access to unhealthy foods, our results show an increase in the consumption of “comfort food”. Such food consists of chocolate, sweet packaged snacks, ice cream and desserts, but above all there was an increase in consumption of pasta and rice, as well as bread, pizza and bakery products (Figure 2). We also found a greater increase the intake of milk, cheese, yogurt, meat, fish, eggs and processed meat (Figure 2). In the first lockdown, approximately 16% of the children and adolescents analyzed had a body weight gain of more than 3 kg. There was a greater weight gain in adolescents than children (67% vs. 55%, *p* = 0.01, respectively), but there was no difference between genders (63% vs. 56%, *p* = 0.17, respectively). We found that neither the basal body weight nor the basal BMI correlated with the body weight gain.

Eating habits are different between children and adolescents [21]. In particular, we found that the weight gain in children was positively associated with a change in body height, and an increase in the intake of milk, cheese, yogurt and sweet packaged snacks. However, the weight gain in adolescents was positively associated with an increased consumption of processed meat, bread, pizza and bakery products (Table 3).

Our results are in line with other studies [15,22,23] and with a previous study by our research group on young university students in Italy [4]. Increases in comfort food consumption and body weight could be related to an increase in boredom at home; children may have tried to help pass the time by eating and found comfort in food during this period.

Our results agree with a recent study that showed that increased boredom in children was strongly related to increased food responsiveness, increased emotional overeating and increased snack frequency, even in young children [24]. According to this research, children rely less on their internal signals for food intake [25] and it is therefore important to encourage children to interpret the signals from the body and to control food intake even in challenging situations, such as in the lockdown. Other factors may have increased the intake of comfort foods during the COVID-19 lockdown, including more sedentary time at home, watching television during meals and other habit changes [26]. In fact, it was found that during the COVID-19 lockdown, exposure to electronic devices increased by as much as four hours a day among children and adolescents [15]. The uncertainty caused by COVID-19 may have led families to change food habits and the environment at home. One study reported that families with food insecurity increased the quantity of high-calorie foods, increased the disposability of non-perishable processed foods in their homes and exerted greater pressure to children to eat [27]. Physical activity was also decreased; with recreational areas, parks and sports clubs closed, there has been further reductions in physical activity in children and adolescents [28] and our data agree with this phenomenon.

Fortunately, our results show an increase in fresh fruit, legume, vegetable and fish consumption, and we also found a reduction in the intake of butter, margarine and oils, and, in a small group of subjects, decreased consumption of candy and sweetened beverages. Our findings are supported by some authors who found an increased intake of these kinds of food in 820 adolescents during the first lockdown [22]. However, this result is not sufficient to prevent the risk associated with weight gain.

In line with other research [4,15,22,23], our study shows that, compared to those with a stable/reduced body weight, subjects with an increased body weight had worsened eating habits.

Interestingly, body weight gain was associated with the consumption of different foods between children and adolescents, reflecting different food choices, preferences and food responsiveness. The differences in food consumption that we found between children and adolescents could be explained by less parental control and greater autonomy in the food choices of the latter [29]. However, other factors could also have influenced the eating behavior of children and adolescents during the first lockdown, such as individual differences, feelings of hunger and satiety, mood state and environment/context [30].

Nutritional choices during childhood and adolescence are very important for growth [31], and the development of lifelong eating behaviors [32] and may have long-term health implications. In fact, being overweight and obese in childhood and adolescence leads to a high risk of health conditions in adulthood [33]. It is thus important to evaluate changes in dietary habits by age due to lockdowns in order to identify young people at risk of increasing their body weight. Obesity has in fact been identified as a risk factor for COVID-19 disease severity [34]. A recent study reported high rates of SARS-CoV-2 infection among children living in high-risk places, presenting asymptomatic or mild disease [35], therefore, weight gain in children and adolescents could aggravate COVID-19 outcomes. Of note, the basal BMI did not correlate with the body weight gain. This is in line with a recent meta-analysis [36]. In fact, a BMI significantly higher after/during the lockdown period in individuals of all ages was observed [36]. An analysis of subgroups, after excluding obese subjects, did not modify this finding [36].

The strengths of our study include the online survey, which allowed us to quickly reach a sufficiently large sample of parents of children and adolescents, both in northern and southern Italy, during a period marked by strong restrictions on movement. Internet penetration stood at 83.7% in January 2021 in Italy [35] which contributed to the large number of people taking part in this survey [37].

Our study has limitations: first, we used self-reported data, such as weight and height, which could lead to under/over-estimation of the weight change; however, our survey was similar to others that have been frequently employed [23]. It is well accepted that when performing a survey across a country (as in our study in which we enrolled individuals from both the regions of Calabria and Lombardy in the south and north of Italy, respectively), the researchers would not have the time and funding to travel to each participant in the study and take reliable measurements, such as anthropometrics. A valid solution to this is through accurate self-reported measures [38]. Furthermore, our method is consistent with a recent study [39].

A recent approach used in surveys of food habits is the Internet-based dietary intake assessment [40]. Thanks to this new approach, it is possible to assess the dietary intake of adolescents by self-administration in a broad international context [41]. In fact, it has been reported that adolescents might be more motivated to report their dietary intake with computer use [42]. Self-administered computerized assessment is a valid way of collecting data [41]. It makes it possible for participants (adolescents as well as the children’s parents) to assess their dietary intake at their convenience, saving considerable time and decreasing costs [41].

A potential limitation of our method was that parents/caregivers may not have been fully aware of all the food items eaten by their children, especially foods eaten outside of the home. Obviously, this was not the case due to the lockdown. A recent meta-analysis which included several studies carried out with children aged 5–11 years with dietary intake of the children reported by parents/caregivers confirmed the validity of dietary assessment methods in children [43]. In this meta-analysis, child characteristics (including weight status, age and sex) were not found to consistently influence the accuracy of the dietary intake assessment.

In this study, we did not evaluate the consumption of walnuts, almonds and other foods rich in polyunsaturated fatty acids that have positive effects on health or endothelial function [44]. Furthermore, we did not analyze other factors, such as maternal education, the number of family members and the hours spent in front of televisions, electronic games and computers that could have influenced the relationship between body weight gain and increased consumption of certain foods. Finally, we did not collect data on stress during the lockdown.

Nevertheless, we believe that the results of our study are very relevant, because we have demonstrated a nutritional and food transition during the first COVID-19 lockdown, characterized by negative modifications in the eating behaviors of children and adolescents. Inadequate nutrition is one of the major behavioral consequences caused by the current COVID-19 pandemic. Since childhood and adolescence are essential life stages for learning and establishing healthy eating habits, which tend to be maintained later in adult life, our findings suggest that eating habits should be closely monitored to avoid the establishment of unhealthy eating habits and prevent body weight gain in children and adolescents while the COVID-19 pandemic is still ongoing. Furthermore, an increase in body weight can also promote psychological changes such as dissatisfaction with body image, low self-esteem [45] and depressive symptoms [46].

Our study highlights that in order to prevent obesity and other related diseases, parents, children and adolescents need to be made aware of the most important principles of healthy nutrition, especially during situations of high stress, such as the COVID-19 pandemic.

## 5. Conclusions

In this study, we have provided, for the first time, data on eating habits by separately analyzing Italian children and adolescents during the COVID-19 lockdown. Children and adolescents are currently considered to be at low risk of falling ill from COVID-19, but this situation may change as obesity increases in this population. The COVID-19 lockdown also caused negative changes in nutrition habits, and weight gain was associated with dietary changes. Furthermore, the increased rates of overweight and obesity in children and adolescents could worsen COVID-19 outcomes and promote an increased risk of developing psycho-social problems. In summary, these results suggest the urgent need for nutrition education strategies to counter the onset of unhealthy eating habits and increasing obesity and other related diseases, especially in children and adolescents.

## Figures and Tables

**Table 1:** Demographic and anthropometric characteristics and lifestyle habit changes of children and adolescents during the first COVID-19 lockdown.

Characteristics	*n*	%
Gender		
Boys	246	56
Girls	193	44
Age groups		
5–9 (years)	255	58.1
10–14 (years)	184	41.9
Weight changes during the quarantine		
Gained	262	59.7
No changes	155	35.3
Reduced	22	5.0
Physical activity during the quarantine		
More sedentary lifestyle	349	79.5
Less sedentary lifestyle	44	10
Did not know	46	10.5

**Table 2:** Variation of anthropometric characteristics, lifestyle and eating habits of children and adolescents according to the change in body weight during the first COVID-19 lockdown.

	Children (5–9 Years)			Adolescents (10–14 Years)		
	Not Increased BW ^a^ (*n* = 116)	Increased BW (*n* = 139)	*p*	Not Increased BW ^a^ (*n* = 61)	Increased BW (*n* = 123)	*p*
Age (years)	6.6 ± 2	7.1 ± 1	0.003	11.3 ± 1	11.7 ± 1	0.06
Boys (%)	53	57	0.52	51	61	0.20
Weight (kg)	26.6 ± 10	28.9 ± 12	0.14	35.3 ± 13	39.2 ± 13	0.10
Δ Weight (kg)	−0.27 ± 1	2.60 ± 2	<0.001	−0.78 ± 1	2.98 ± 3	<0.001
Height (cm)	119 ± 13	121 ± 12	0.28	139 ± 18	149 ± 14	0.06
Δ Height (cm)	2.1 ± 3	4.2 ± 3	<0.001	2.8 ± 3	3.3 ± 3	0.54
BMI (kg/m^2^)	16 ± 3	16.5 ± 3	0.18	18.5 ± 5	19.7 ± 4	0.10
Δ BMI (kg/m^2^)	−0.6 ± 0.8	0.5 ± 1	<0.001	−1.2 ± 1	0.6 ± 0.9	<0.001
More sedentary lifestyle (%)	85	88	0.58	79	95	0.001
Eating Habit Change (Increase in intake, %)						
Milk, cheese and yogurt	5	26	<0.001	10	23	0.043
Meat, fish and eggs	13	18	0.30	8	18	0.12
Processed meat	13	38	<0.001	12	28	0.014
Pasta and rice	14	34	<0.001	18	24	0.45
Bread, pizza and bakery products	42	58	0.017	30	49	0.017
Vegetables, legumes and fruits	20	26	0.29	15	12	0.64
Oil, butter and margarine	5	14	0.033	5	8	0.54
Sweet beverages	8	13	0.22	3	14	0.037
Ice cream and desserts	24	45	0.001	19	33	0.08
Sweet packaged snacks	23	46	<0.001	23	37	0.06
Candy	16	26	0.037	15	26	0.09
Chocolate	23	44	0.001	20	33	0.08

Data are given as mean ± SD or prevalence as appropriate. ^a^ “Not increased body weight” means either stable or decreased; Δ, change.

**Table 3:** Logistic regression analysis: dietary changes and other factors associated with body weight increase according to age group during the first COVID-19 lockdown.

Children (5–9 Years)						
Dependent Variable					95% CI	
Increased BW *	B	SE	*p*	OR	LL	UL
Δ Height	0.38	0.09	<0.001	1.47	1.21	1.78
Milk, cheese and yogurt	1.67	0.55	0.002	5.33	1.81	15.70
Sweet packaged snacks	1.06	0.36	0.004	2.90	1.41	5.96
**Adolescents (10–14 Years)**						
Dependent variable					95% CI	
Increased BW ^§^	B	SE	*p*	OR	LL	UL
Processed meat	1.02	0.50	0.04	2.79	1.04	7.44
Bread, pizza and bakery products	0.79	0.40	0.05	2.21	0.99	4.92

* Excluded variables: age, pasta/rice, bread/pizza/bakery products, oil/butter/margarine, ice cream/dessert and candy. ^§^ Excluded variables: age, physical activity change, baseline height, milk/cheeses/yogurt, meat/fish/eggs, sweet beverages, ice cream/dessert, sweet packaged snacks, chocolate and candy. B, unstandardized coefficient; SE, standard error; OR, odds ratio; CI, confidence interval; LL, lower limit; UL, upper limit.

## Data Availability

The data presented in this study are available on request from the corresponding author.
